# The relationship between type of urinary diversion and quality of life after radical cystectomy: Ileal conduit versus orthotopic bladder

**DOI:** 10.1002/bco2.29

**Published:** 2020-06-19

**Authors:** Mohamed S. Elbadry, Ahmed Issam Ali, Ali Hassan, Kieran David Clement, Ahmed Rashed Hammady, Abdalla Abdbelaal, Nady Mounir Barsoum, Mohamed Abd Elmalek Hassan, Ahmed H. Gabr

**Affiliations:** ^1^ Department of Urology Minia University Minia Egypt; ^2^ NHS Greater Glasgow and Clyde (NHSGGC) Glasgow UK; ^3^ Department of Urology Faculty of Medicine Sohag University Sohag Egypt; ^4^ Department of Urology Ain Shams University Cairo Egypt; ^5^ Department of Urology Royal Alexandra Hospital Paisley UK

**Keywords:** bladder neoplasm, ileal conduit, orthotopic diversion, quality of life, radical cystectomy

## Abstract

**Objectives:**

We aimed to compare health‐related quality of life (HrQoL) in patients who underwent ileal conduit (IC) vs orthotopic neobladder (ONB) as a method of urinary diversion (UD) after radical cystectomy (RC) for invasive bladder cancers.

**Methods:**

The questionnaires of the Functional Assessment of Cancer Therapy‐Bladder Cancer (FACT‐BL) were used to evaluate and compare the HrQoL in 113 patients with 1 year follow‐up.

**Results:**

Forty‐nine patients were included in the ONB group and 64 patients in the IC group. Patients with IC showed superior scores in all domains of the FACT‐BL questionnaire and this reached statistical significance in physical well‐being (PWB), functional well‐being (FWB), over all FACT‐G, Bladder‐Specific Subscale and FACT‐BL total scores (*P*‐values = .01, .01, .001, .001, and .001, respectively).

**Conclusions:**

Our findings demonstrate marginally improved HrQoL in IC patients when compared with patients undergoing ONB which may be attributed to an increased morbidity and postoperative complications in the ONB group.

## 1 INTRODUCTION

Radical cystectomy (RC) and urinary diversion (UD) with or without neoadjuvant chemotherapy is considered the gold standard treatment for muscle invasive bladder cancer.^1^, ^2^

The technique employed for UD following cystectomy is typically through surgeon choice and patient preference with the two commonest employed techniques being an ileal conduit (IC) or orthotopic neobladder (ONB). However, many centers worldwide argue believe that ONB should be the default choice^3^ Despite this, there are associated metabolic, physical, and functional considerations such as urinary function and sexual activity that can affect a patients’ quality of life (QoL) following cystectomy. These factors are central in deciding which type of UD will be performed.^4^

The above changes associated with UD affecting a patient's QoL are difficult to measure.^5^ Health‐related quality of life (HrQoL) in patients with various types of UD has been evaluated by several studies, but without a definitive conclusion.^5^

The aim of the present study is to compare HrQoL between patients who underwent ONB or IC following RC using a validated bladder cancer‐specific questionnaire.

## 2 PATIENT AND METHODS

Through a prospective, observational, comparative, hospital‐based study, patients who underwent RC with UD (either IC or ONB) were included in our study in the period between September 2015 and November 2018. The shared‐decision to utilize one method of reconstruction over another was made between the surgeon and patient following a discussion of the benefits and risks of each modality. All cystectomies were performed using an open technique and ONB were performed using a consistent method utilized in our center. Patients in both groups were followed up routinely at 3, 6, and 12 months postoperatively.

Our exclusion criteria were the presence of metastatic or recurrent disease, patients’ preference to not be included in the study, missed preoperative data, patients who died and patients who received palliative therapy during the 12 month follow‐up period.

Our demographic data included age, sex, body mass index (BMI), comorbidities, pathological tumor stage, histopathology of the specimen, postoperative complications, and adjuvant therapy undertaken.

For assessment of QoL, we utilized the validated Arabic version of the Functional Assessment of Cancer Therapy‐Bladder Cancer (FACT‐BL) Questionnaire version 4 which was completed by patients’ at 1 year following surgery. The English version of the questionnaire is shown in Appendix 1.

The FACT‐BL questionnaire is designed to evaluate QoL in patients with bladder cancer and consists of two parts. The first part is the general version (FACT‐G) assessing four domains (physical well‐being, social/family well‐being, emotional well‐being, and functional well‐being), with patients responding to statements on a five‐level ordinal Likert Scale, ranging from “not at all” to “very much.” Higher scores indicate a better QoL.

The second part is a bladder cancer‐specific module. It evaluates urinary, intestinal, and sexual function, with a total of 10 statements, plus two more for patients with a stoma.

The questions are scored based on a scale from 0 to 4, with 0 being not at all and 4, very much, with the higher the score again indicating a better QoL.^6^ The questionnaires were completed by participants in clinic 12 months postoperatively.

### 2.1 Sample size

A study sample size was calculated using G* power software version 3.1.9.4. Test family (*t* tests), type of power analysis (A priori: Compute required sample size—given α, power and effect size), input parameters, effect size = 0.64, α error = 0.05, power (1 − β) = 0.8, and with assuming allocation ratio N1/N2 = 1. The resulting output parameters provided a total sample size of 80 patients with 40 patients in each group.

### 2.2 Statistical analysis

All statistical calculations were done using computer programs SPSS (Statistical Package for the Social Science; SPSS Inc., Chicago, IL, USA) version 19 for Microsoft Windows. Differences in the mean of continuous variables were analyzed using parametric test (Paired sample *t* test) for variables before and after intervention in the same group while the independent sample *t* test was used to compare variables in between the two study groups. Differences between categorical variables were analyzed using the Chi‐Square test. The associations between continuous variables were determined using Pearson Product‐Moment Correlation while associations between categorical variables were assessed in each group by independent sample *t* test. For all tests, a *P*‐value of < .05 was regarded as statistically significant.

## 3 RESULTS

137 patients who underwent RC between September 2015 and November 2018 were included in our study. Three patients refused to participate in the study, six patients with missing preoperative data were excluded, seven patients died during the postoperative follow‐up period, five patients developed local recurrence, and three patients developed distant metastasis, and therefore, were excluded from the study. The final analysis included 64 patients in the IC group and 49 in the ONB group who had available follow‐up for 12 months.

There was no significant difference between the groups in terms of age, BMI, sex, pathological tumor stage, and adjuvant therapy (Table 1).

**Table 1:** Distribution of selected demographic and pathological variables among the study groups

Demographic and clinical variables	Types of diversion		*P*‐Value
	ONB (*n* = 49)	Ileal conduit (*n* = 64)	
Age (yrs)	59.3 ± 8.3	61.9 ± 7	.1
Gender			
Male	44 (89.8%)	53 (82.8%)	.4
Female	5 (10.2%)	11 (17.2%)	
BMI (kg/m^2^)	28 ± 6.5	27.1 ± 5.3	.8
Histopathology, *n* (%)			.05*
TCC	36 (73.5%)	58 (90.6%)	
SCC	11 (22.4%)	5 (7.8%)	
Adenocarcinoma	2 (4.1%)	1 (1.6%)	
Pathological tumor stage, *n* (%)			.07
T1	9 (18.4%)	20 (31.3%)	
T2	19 (38.7%)	12 (18.8%)	
T3	21 (29.9%)	29 (45.3%)	
T4a	0	3 (4.7%)	
Adjuvant chemotherapy given, *n* (%)	12 (24.5%)	22 (34.4%)	.2

**Figure 1:**
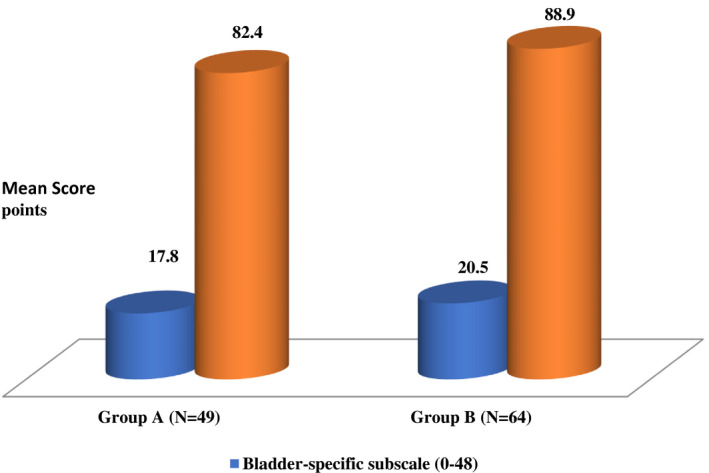
Bladder‐specific scale and total FACT‐BL scores. Group A: ONB (N = 49) Group B: (N = 64)

Patients undergoing IC showed higher scores in all domains than ONB patients in physical well‐being (PWB), functional well‐being (FWB), over all FACT‐G score, Bladder‐specific subscale score and FACT‐BL total score (*P*‐value = .01, .01, .001, .001, and .001, respectively). These data are demonstrated in Table 2 and Figure 1.

**Table 2:** Quality of life (QOL) scores for both groups

Domain (Score range)	ONB (N = 49)	IC (N = 64)	*P*‐value
PWB (0‐28)	13.7 ± 1.4	15 ± 1.6	.01*
SWB (0‐28)	21.8 ± 1.8	21.7 ± 1.8	.7
EWB (0‐24)	14.5 ± 4	15.3 ± 3.9	.2
FWB (0‐28)	14.4 ± 3.2	16.2 ± 3.3	.01*
FACT G	64.6 ± 5	68.3 ± 5.7	.001*
Bladder‐specific subscale (0‐48)	17.8 ± 5.8	20.5 ± 4.9	.001*
FACT‐BL total score (0‐156)	82.4 ± 7.4	88.9 ± 7.7	.001*

No significant difference was recorded between FACT‐BL total score and age, sex, and comorbidities as shown in Table 3.

**Table 3:** Comparison between FACT‐BL score and patients’ age, sex, and comorbidities

		ONB (n = 49)		IC (n = 64)	
		Total score		Total score	
		Mean ± SD	*P*‐value	Mean ± SD	*P*‐value
Age groups					
<60 years		83.5 ± 7.1	.3	88.4 ± 8.2	.6
>60 years		81.5 ± 7.7		89.3 ± 7.3	
		**Group A (n = 49)**		**Group B (n = 64)**	
Sex					
Male		82.2 ± 7.3	.4	89.5 ± 7.6	.1
Female		84.4 ± 9.3		85.9 ± 8	
		**ONB (n = 49)**		**IC (n = 64)**	
Comorbidities					
Previous abdominal surgery	No	82.7 ± 8	.4	88.4 ± 7.8	.5
	Yes	81.4 ± 5.3		89.8 ± 7.6	
DM	No	81.8 ± 7.6	.3	88.8 ± 7.5	.8
	Yes	84 ± 7.1		89.4 ± 9.6	
HTN	No	82.8 ± 7.7	.4	89.4	.1
	Yes	81 ± 6.3		85.1	
Heart disease	No	82.5 ± 7.5	.5	88.7 ± 7.5	.5
	Yes	79.5 ± 4.9		91.5 ± 5	
Hepatic disease	No	83.3 ± 8.3	.4	88.8 ± 7.5	.8
	Yes	81.6 ± 6.6		89 ± 8	
COPD	No	82.8 ± 7.4	.2	88.4 ± 8	.3
	COPD	76 (No SD)		91.7 ± 5.6	
CKD (cr. ≥ 3 mg/dL)	Free	82.3 ± 7.5	.7	88.7 ± 8.2	.6
	CKD	83.6 ± 7.6		89.7 ± 5.5	
Stroke	No	82.4 ± 7.5	.9	88.9 ± 7.7	.9
	Yes	82 ± 7		89.3 ± 10.6	

In ONB group, early postoperative complications included wound complication in 21 cases (42.9%), urinary leakage in 14 cases (28.6%), ileus in 14 cases (28.6%), and venous thromboembolism in 3 cases (6.1%). Regarding late postoperative complications; nocturnal incontinence was found in nine cases (18.4%), chronic urine retention in three cases (6.1%), ureteroileal stricture in two cases (4.1%), ureterroileal stricture in one case (2%), recurrent UTIs in three cases (6.1%), and incisional hernia in four cases (8.2%).

In IC group, early postoperative complications included wound complication in 33 cases (51.6%), urinary leakage in 6 cases (9.4%), ileus in 12 cases (18.8%), and thromboembolic complication in 6 cases (9.4%). Regarding late postoperative complications, ureteroileal stricture was found in three cases (4.7%), recurrent UTIs in nine cases (14.1%), and incisional hernia in three cases (4.7%).

In patients undergoing ONB, FACT‐BL total score, and postoperative complications were found to be significantly correlated (*P* = .04). This was not the case for the IC group (*P* = .6) as shown in Table 4.

**Table 4:** Comparison between postoperative complication and total FACT‐BL score in both groups (Using Clavien‐Dindo score)

Postoperative complications	ONB (n = 49)		IC (n = 64)	
	Total score		Total score	
	Mean ± SD	*P*‐value	Mean ± SD	*P*‐value
Major complications (≥3)	81.3 ± 7.1	.04*	88.4 ± 7.9	.6
Minor complications (<3)	86.7 ± 7.3		89.3 ± 7.3	

## 4 DISCUSSION

There are many techniques described for reconstruction of the lower urinary tract and trends in the way urologists select the type of urinary reconstruction as the most appropriate UD after RC are changing.^2^

Clearly it is important to assess patients’ QoL to understand the psychological, social, and physical consequences of the various types of urinary reconstruction. The comparative impacts among different types of UDs on HrQoL have not been well studied. Postoperatively patients may struggle with surgery‐related problems including, but not limited to, urine leakage, change of body image, and loss of sexual interest. Various studies have assessed the QoL of bladder cancer patients using validated questionnaires nonspecific to bladder cancer.^4^, ^7^ FACT‐BL, a bladder‐cancer‐specific questionnaire has recently become available for use in conjunction with FACT‐G for this use.^6^ To the authors’ knowledge, few studies have compared the QoL of patients with an IC or an orthotropic neobladder (ONB), using the FACT‐BL questionnaire.^8^

In the present study, the Arabic version of the FACT‐BL was used to assess for potential differences in QoL among patients with bladder cancer after RC with IC or ONB reconstruction. At 1 year postoperatively, we found that QoL scores are higher (indicating superiority) in IC patients when compared with ONB patients, which could be explained by a higher postoperative morbidity associated with reconstruction using an ONB. These results are similar to those of Anderson and colleagues who reported higher QoL at 1 year postoperatively in IC UD patients compared to neobladder patients.^9^ Another study by Gellhaus et al reported a higher QoL scores among IC patients compared to neobladder patients more than 10 years after UD.^10^ Conversely, Hobisch et al, demonstrated higher scores of HrQoL in ONB patients compared to IC patients.^7^ Similarly, in their prospective study, Singh et al, concluded a better physical and social function among ONB compared to patients who underwent an IC.^11^

A multicenter Italian study investigated the QoL in neobladder patients using the IONB‐PRO and EORTC QLQ‐BLM30 questionnaires reported that longer follow‐up and lack of urinary incontinence were predictors of better emotional and relational health. These results are limited, however, in lacking baseline data and a comparative group.^12^

A systematic review by Ghosh et al suggested that improved surgical techniques representative in modern comparison studies favor ONB in terms of higher QoL scores after RC.^13^ In contrast, a large meta‐analysis of observational HrQoL studies that using validated questionnaires found no statistically significant difference in HrQoL scores between ONB or IC patients. However, QoL outcomes were significantly better among patients treated with an ONB in a sub‐analysis of studies based on the EORTC QLQ‐C30 questionnaire.^14^

Dutta et al used FACT‐G in 72 patients with an IC or ONB and found no significant differences in total FACT‐G score between the two groups.^9^ However, they noted that patients with an ONB had a significantly better QoL than those with an IC in the areas of EWB and FWB.

In our study, IC patients showed better scores in all domains than those who underwent ONB reconstruction and this reached statistical significance in PWB, FWB, over all FACT‐G, Bladder‐specific subscale and FACT‐BL total score. In contrast, a study done by Kikuchi et al did not find any difference between the two groups in any of four domains of FACT‐G.^15^

Mansson et al compared the QoL of 64 patients with a Continent Reservoir (CR) or ONB using FACT‐BL and observed no differences in any domain of FACT‐G between the two groups.^6^ In their study, patients with a continent reservoir had significantly less trouble controlling urine as would be expected, and patients with an ONB had a significantly better appreciation of their body appearance.

Urinary troubles after RC and urinary tract reconstruction have a significant impact on patients’ HrQoL.^16^ Thulin et al observed more nocturnal urinary leakage and frequency in ONB patients, which subsequently affected patients’ sleep and decreased their HrQoL compared to other diversion methods. This resulted in lower self‐assessed HrQoL, physical health, and energy levels.^17^ Protogerou et al, reported that IC had a worse effect on the urinary functions.^18^ Similarly, other studies have also demonstrated poorer urinary function (urinary leakage and lack of urinary control) in ONB compared to IC.^19^, ^20^

In the current study, the Bladder cancer subscale (BLCS) of FACT‐BL Score, which assesses the urinary and sexual function, body weight and bowel habits, was significantly higher in IC patients. This may be explained by use of better stoma appliances, and postoperative care by an experienced and skilled stoma therapist. Some degree of urinary leakage, especially at night, is a common finding in ONB patients. Patients with an IC may come to manage their diversion better over time and this situation may make urine control more easily manageable and their QoL may then be comparable to that of those with an ONB.

Our study is limited as we do not have access to a base line assessment of QoL in order to compare preoperative and postoperative assessments. Furthermore, we have not adjusted for the effects of chemotherapy on HrQoL in patients who received adjuvant chemotherapy. Finally, our follow‐up is limited to 1 year and it is possible that further improvements in HrQoL may be seen over longer time periods as has been demonstrated in other studies, particularly for patients with an ONB.

## 5 CONCLUSIONS

Based on our prospective, observational, comparative, hospital‐based study with 1 year follow‐up, patients with an IC UD showed marginally better QoL scores when compared with patients with an ONB.

However, further multicenter studies with larger sample sizes and longer‐term follow‐up periods may be required to determine subtle differences between both techniques.

## STATEMENT OF ETHICS

All procedures performed were in accordance with the ethical standards of the institutional and/or national research committee. All subjects had given informed consent.

## DISCLOSURE STATEMENT

The authors have no conflicts of interest to declare.

## AUTHOR CONTRIBUTIONS

All authors have made a significant contribution to the findings and methods in the paper. All authors have read and approved the final draft.

## Supporting information

Supplementary Material
