# Supplementary material for: The relationship between type of urinary diversion and quality of life after radical cystectomy: Ileal conduit versus orthotopic bladder
Source: BJUI Compass. 2020 Jun 19;1(4):133–8. doi: 10.1002/bco2.29 (PMC8988837; doi:10.1002/bco2.29)
Supplement: Supplementary file 1 — Supplementary Material [file BCO2-1-133-s001.docx]

- **Appenix 1: Questionnaire design and questions:**

| **Domain** | | **Reverse item** | **Scale from 1-4 points** | | | | | **Individual score** |
| --- | --- | --- | --- | --- | --- | --- | --- | --- |
| **PHYSICAL WELL-BEING (PWB)** | |  | **Not at all** | **A little bit** | **Some-what** | **Quite a bit** | **Very much** |  |
| GP1 | I have a lack of energy | - | 0 | 1 | 2 | 3 | 4 |  |
| GP2 | I have nausea | - | 0 | 1 | 2 | 3 | 4 |  |
| GP3 | Because of my physical condition, I have trouble meeting the needs of my family | - | 0 | 1 | 2 | 3 | 4 |  |
| GP4 | I have pain | - | 0 | 1 | 2 | 3 | 4 |  |
| GP5 | I am bothered by side effects of treatment | - | 0 | 1 | 2 | 3 | 4 |  |
| GP6 | I feel ill | - | 0 | 1 | 2 | 3 | 4 |  |
| GP7 | I am forced to spend time in bed | - | 0 | 1 | 2 | 3 | 4 |  |
| Sum of individual score | | | |  | | | | |
| Subscale score  **(multiply sum by total number of questions then divide by actual number answered** | | | | |  | | | |

| **Domain** | | **Reverse item** | **Scale from 1-4 points** | | | | | **Individual score** |
| --- | --- | --- | --- | --- | --- | --- | --- | --- |
| **SOCIAL WELL-BEING (SWB)** | |  | **Not at all** | **A little bit** | **Some-what** | **Quite a bit** | **Very much** |  |
| GS1 | I feel close to my friends | + | 0 | 1 | 2 | 3 | 4 |  |
| GS2 | I get emotional support from my family | + | 0 | 1 | 2 | 3 | 4 |  |
| GS3 | I get support from my friends | + | 0 | 1 | 2 | 3 | 4 |  |
| GS4 | My family has accepted my illness | + | 0 | 1 | 2 | 3 | 4 |  |
| GS5 | I am satisfied with family communication about my illness | + | 0 | 1 | 2 | 3 | 4 |  |
| GS6 | I feel close to my partner (or the person who is my main support | + | 0 | 1 | 2 | 3 | 4 |  |
| Q1 | *Regardless of your current level of sexual activity, please answer the following question. If you prefer not to answer it, please mark this box and go to the next section.* | | | | | | | |
| GS7 | I am satisfied with my sex life | + | 0 | 1 | 2 | 3 | 4 |  |
| Sum of individual score | | | |  | | | | |
| Subscale score  **(multiply sum by total number of questions then divide by actual number answered** | | | | |  | | | |

| **Domain** | | **Reverse item** | **Scale from 1-4 points** | | | | | | **Individual score** |
| --- | --- | --- | --- | --- | --- | --- | --- | --- | --- |
| **EMOTIONAL WELL-BEING (EWB)** | |  | **Not at all** | **A little bit** | **Some-what** | | **Quite a bit** | **Very much** |  |
| GE1 | I feel sad | - | 0 | 1 | 2 | | 3 | 4 |  |
| GE2 | I am satisfied with how I am coping with my illness | + | 0 | 1 | 2 | | 3 | 4 |  |
| GE3 | I am losing hope in the fight against my illness | - | 0 | 1 | 2 | | 3 | 4 |  |
| GE4 | I feel nervous | - | 0 | 1 | 2 | | 3 | 4 |  |
| GE5 | I worry about dying | - | 0 | 1 | 2 | | 3 | 4 |  |
| GE6 | I worry that my condition will get worse | - | 0 | 1 | 2 | | 3 | 4 |  |
| Sum of individual score | | | |  | | | | | |
| Subscale score  **(multiply sum by total number of questions then divide by actual number answered** | | | | | |  | | | |

| **Domain** | | **Reverse item** | **Scale from 1-4 points** | | | | | **Individual score** |
| --- | --- | --- | --- | --- | --- | --- | --- | --- |
| **FUNCTIONAL WELL-BEING (FWB)** | |  | **Not at all** | **A little bit** | **Some-what** | **Quite a bit** | **Very much** |  |
| GF1 | I am able to work (include work at home) | + | 0 | 1 | 2 | 3 | 4 |  |
| GF2 | My work (include work at home) is fulfilling | + | 0 | 1 | 2 | 3 | 4 |  |
| GF3 | I am able to enjoy life | + | 0 | 1 | 2 | 3 | 4 |  |
| GF4 | I have accepted my illness | + | 0 | 1 | 2 | 3 | 4 |  |
| GF5 | I am sleeping well | + | 0 | 1 | 2 | 3 | 4 |  |
| GF6 | I am enjoying the things I usually do for fun | + | 0 | 1 | 2 | 3 | 4 |  |
| GF7 | I am content with the quality of my life right now | + | 0 | 1 | 2 | 3 | 4 |  |
| Sum of individual score | | | |  | | | | |
| Subscale score  **(multiply sum by total number of questions then divide by actual number answered** | | | | |  | | | |

| **Domain** | | **Reverse item** | **Scale from 1-4 points** | | | | | **Individual score** |
| --- | --- | --- | --- | --- | --- | --- | --- | --- |
| **PHYSICAL WELL-BEING (PWB)** | |  | **Not at all** | **A little bit** | **Some-what** | **Quite a bit** | **Very much** |  |
| BL1 | I have trouble controlling my urine | - | 0 | 1 | 2 | 3 | 4 |  |
| C2 | I am losing weight | - | 0 | 1 | 2 | 3 | 4 |  |
| C3 | I have control of my bowels | + | 0 | 1 | 2 | 3 | 4 |  |
| BL2 | I urinate more frequently than usual | - | 0 | 1 | 2 | 3 | 4 |  |
| C5 | I have diarrhea (diarrhoea) | - | 0 | 1 | 2 | 3 | 4 |  |
| C6 | I have a good appetite | + | 0 | 1 | 2 | 3 | 4 |  |
| C7 | I like the appearance of my body | + | 0 | 1 | 2 | 3 | 4 |  |
| BL3 | It burns when I urinate | - | 0 | 1 | 2 | 3 | 4 |  |
| BL4 | I am interested in sex | + | 0 | 1 | 2 | 3 | 4 |  |
| BL5 | (For men only) I am able to have and maintain an erection | + | 0 | 1 | 2 | 3 | 4 |  |
| Q2 | Do you have an ostomy appliance? No___ Yes___ If yes, answer the following two item | | | | | | | |
| C8 | I am embarrassed by my ostomy appliance | - | 0 | 1 | 2 | 3 | 4 |  |
| C9 | Caring for my ostomy appliance is difficult | - | 0 | 1 | 2 | 3 | 4 |  |
| Sum of individual score | | | |  | | | | |
| Subscale score  **(multiply sum by total number of questions then divide by actual number answered** | | | | |  | | | |
